# How far can you borrow? Divergence limits of cross-species Gene Ontology enrichment in non-model organisms

**DOI:** 10.1093/bib/bbag622

**Published:** 2026-11-09

**Authors:** Sung-Gwon Lee, Chungoo Park

**Affiliations:** Biomedical Research Institute, Chonnam National University Hospital, Baekseo-ro 160, Dong-gu, Jeonnam-Gwangju 61469, Republic of Korea; School of Biological Sciences and Technology, Chonnam National University, Yongbong-ro 77, Jeonnam-Gwangju 61186, Republic of Korea; Institute of Systems Biology & Life Science Informatics, Chonnam National University, Yongbong-ro 77, Jeonnam-Gwangju 61186, Republic of Korea

**Keywords:** gene ontology, enrichment analysis, non-model organisms, orthology, functional annotation transfer, phylogenetic distance, benchmark

## Abstract

Functional interpretation of omics data from non-model species almost always relies on borrowing Gene Ontology (GO) annotations from a model organism—often a distant one (*Arabidopsis* for plants, zebrafish or human for fish)—and running enrichment analysis on inferred orthologs. How far this practice can be pushed before the enrichment result degrades has never been quantified at the level practitioners use. We present the first systematic benchmark of the divergence-tolerance of cross-species GO-transfer enrichment, across six focal species spanning three eukaryotic kingdoms, each with 15–22 reference species tiling a continuous phylogenetic gradient. Set-level fidelity—measured as redundancy-robust Wang semantic similarity between transferred and experimental-truth enriched term sets—declines monotonically with distance, collapsing to chance at the clade boundary in some panels (fish, rice) while remaining well above it in others (insect, fungi, mammal) (half-collapse identity, ID50, ~40%–59%). At fixed distance, a richer reference raises recall but lowers precision, quantifying a long-recognized but unresolved trade-off. Conserved processes transfer reliably to distant references whereas taxon-specific processes do not, a pattern consistent across all six focal species tested. Whole-genome duplication degrades transfer only when ohnolog retention is high, showing up as sensitivity to the orthology method rather than a steeper decline. An independent, GO-free measure of expression conservation supports the curves in vertebrates. We condense these results into a focal-species × distance reliability guide and a step-by-step decision protocol that let researchers judge, before analysis, whether borrowed-GO enrichment can be trusted for their species and question.

## Introduction

Next-generation sequencing has made transcriptomes and genomes routine for essentially any organism, but turning these data into biological meaning still depends on functional annotation, and for many species such annotation does not exist [1, 2]. The Gene Ontology (GO) [3, 4] and over-representation (“enrichment”) analysis are the standard route from a gene list to biological interpretation [5–7]. Because the GO is designed to be species-neutral, the near-universal workaround for an unannotated species is to identify putative orthologs in a well-annotated model organism—with tools such as OrthoFinder, OMA, InParanoid or Ensembl Compara [8–11], whose relative accuracy is regularly benchmarked [12]—and transfer that model’s GO terms to the focal genes, then run enrichment as if the species were annotated; plants borrow from *Arabidopsis*, fishes from zebrafish or human, and so on. Pipelines such as Blast2GO, InterProScan and eggNOG-mapper operationalize exactly this transfer [13–17].

The validity of borrowing rests on the *ortholog conjecture*—that orthologs retain ancestral function more faithfully than paralogs [18, 19]—which is empirically debated: large-scale analyses have variously challenged it [20], defended it after correcting for annotation biases [21], and argued it is untestable with current GO yet supported by expression data [22], a debate recently revisited for function prediction [23]. What these studies share is that they evaluate function at the level of individual gene pairs, as do community function-prediction assessments [24]. The quantity a practitioner obtains, however, is a *set-level* enrichment result, and how faithfully that result survives increasing evolutionary distance has not been systematically measured. Primmer et al. reviewed cross-species GO use and flagged a key tension—a phylogenetically closer reference reduces orthology errors but is often more poorly annotated than a distant but better-curated model—yet quantified neither the tolerance curve nor the trade-off [2].

Here we benchmark cross-species GO-transfer enrichment directly. Treating six well-annotated focal species (mammal, fish, insect, two plants, fungus; three eukaryotic kingdoms) as if they were non-model, we transfer GO from reference species placed along a continuous divergence gradient, run enrichment on controlled gene lists, and compare the borrowed result against each focal species’ own experimental annotation. This lets us (i) chart divergence-tolerance curves and a half-collapse identity (ID50) per clade; (ii) disentangle reference distance from reference annotation richness; (iii) resolve how GO aspect and term specificity shape transferability; (iv) test the effect of orthology-assignment method and whole-genome duplication; (v) validate the signal with a GO-independent expression-conservation measure; and (vi) condense the results into a practical reliability guide. Overall, the decline has the same shape in every clade but sets in at different distances, and the type of function asked about (conserved versus taxon-specific) and the reference chosen (distance versus annotation richness) matter as much as raw distance.

## Materials and methods

### Benchmark design and divergence axis

We treated six well-annotated “focal” species—mouse (*Mus musculus*), zebrafish (*Danio rerio*), fruit fly (*Drosophila melanogaster*), rice (*Oryza sativa*), thale cress (*Arabidopsis thaliana*) and budding yeast (*Saccharomyces cerevisiae*), spanning three eukaryotic kingdoms—as if they were unannotated. For each focal species we assembled a panel of 15–22 reference species (111 in total across the six panels) along a continuous gradient of evolutionary distance, from congeneric relatives (which anchor the near-distance plateau) out to deep outgroups (reference species and accessions in Table S1; all six panels are visualized along the divergence axis in Fig. S1). Distance was quantified two ways: divergence time (millions of years) from TimeTree [25], and the median amino-acid identity of one-to-one orthologs computed from the data. All quantitative analyses use the latter, because molecular clocks differ among lineages; divergence time is reported for context only (panel design and species annotations in Table S1 and Fig. S1).

### Proteomes, annotations and the focal “truth”

Reference proteomes (one representative—longest—protein per gene) and GO annotation files were obtained from UniProt [26] and the GO Annotation database, and the go-basic ontology [27] was used for true-path propagation. To avoid validating GO transfer against electronically inferred (homology-based) annotation—a circularity highlighted by [22, 28]—each focal species’ “truth” was restricted to experimental evidence codes—EXP (inferred from experiment), IDA (direct assay), IMP (mutant phenotype), IGI (genetic interaction), IPI (physical interaction), IEP (expression pattern) and their high-throughput equivalents (HTP, HDA, HMP, HGI, and HEP)—and true-path propagated. The reference annotation that is transferred, in contrast, deliberately used each reference’s full annotation (all evidence codes, including electronically inferred terms), because this is what practitioners borrow in real use: transfer pipelines propagate the model organism’s complete GO annotation, not an experimental-only subset. A sensitivity analysis restricting the reference to experimental codes confirmed that this is not a viable alternative practice: reference experimental annotation deep enough to transfer exists for only 30 of the 111 references, and even for those the mean per-reference Wang similarity fell from 0.68 (full annotation) to 0.42 (experimental-only), because the transferred annotation becomes too sparse to reproduce the truth enrichment (per-list results for both evidence sets in Table S2). Circularity is instead prevented by two dedicated safeguards: the experimental-only focal truth described above, and the provenance filter described below.

### Orthology inference and GO transfer

Putative orthologs between focal and reference proteomes were identified with DIAMOND blastp [29] (e-value ≤1 × 10^−5^, query- and subject-coverage ≥50%, `--more-sensitive`, `--max-target-seqs 1`). Three orthology criteria of increasing stringency were compared: best-hit (the single top-scoring reference protein per focal protein), reciprocal-best-hit (RBH; pairs that were mutual top hits in both directions), and eggNOG orthologous-group (OG) co-membership (best-hit pairs whose focal and reference proteins belonged to the same eggNOG group), the last assigned with eggNOG-mapper in DIAMOND mode against the eggNOG 5.0 database [13, 16, 30]. The median amino-acid identity of one-to-one orthologs per focal–reference pair—the cross-clade distance axis—was taken from the DIAMOND alignments. Reference GO terms were transferred to the mapped focal genes; to prevent circular transfer, reference annotations whose provenance traced back to the focal species (by With/From accession or by source taxon) were removed before transfer. Focal genes without an identified ortholog were retained in the gene lists rather than removed: they receive no transferred annotation and—following the standard enricher convention, in which the statistical universe for each aspect comprises the genes carrying at least one annotation in that aspect—they drop out of the transferred-annotation test automatically. This mirrors real practice, where users do not curate gene lists by orthology before running borrowed-GO enrichment; per-reference ortholog counts are given in Table S1, and per-list ortholog-mapping rates in Table S3.

### Gene lists

Two kinds of “list of interest” were tested. Synthetic lists were the member genes of individual focal GO terms (40–250 genes each), balanced across GO aspects and information-content (IC) bins. IC was defined as −log₂(genes annotated to the term ÷ total annotated genes) from the focal truth annotation, and terms were split, within each aspect, into shallow, mid and deep tertiles of IC (low IC = general, high IC = specific); biological-process lists were additionally balanced across the conserved/taxon-specific/other categories defined below. To span the size range of real experiments we additionally built large synthetic lists from general (low-IC) terms, covering ~50–9000 genes. Real differentially expressed gene (DEG) sets were retrieved per experiment and contrast from the EBI Expression Atlas [31] taking significant genes (adjusted *P* < .05 and |log₂ fold-change| ≥ 1), mapping Atlas gene identifiers to focal UniProt accessions and prioritizing contrasts that span diverse experimental factors. The statistical background for every test was the focal species’ measured proteome. A complete inventory of the gene lists—per-focal counts by aspect and IC tertile, size distributions, the conserved/taxon-specific/other composition of the BP lists, and the Expression Atlas experiments and contrasts behind the real DEG sets—is provided in Table S4.

### Enrichment and set-level metrics

Over-representation was computed with a vectorized hypergeometric test (p = P(X ≥ k) for k of the n list genes annotated to a term, from a background of N genes of which M carry that term) with Benjamini–Hochberg false-discovery-rate correction across all tested terms, restricted to term sizes of 5–2000 background genes; a term was called enriched at adjusted *P* < .05. This implementation was validated to be numerically identical to clusterProfiler::enricher [32] (*P*-value correlation 1.000; identical significant sets). For each gene list, enrichment was run once with the focal truth annotation (the reference result) and once with each transferred annotation, and the two significant-term sets were compared. Fidelity was scored in two complementary ways. The primary metric was Wang semantic similarity [33], computed directly from the GO directed acyclic graph: each term’s semantic value is defined over its ancestor graph with the standard semantic-contribution weights (0.8 for *is_a*, 0.6 for *part_of*, as in GOSemSim [34]), the similarity of two terms is the ratio of their shared to total semantic values, and the two significant-term sets (transferred versus truth; the top 50 per list, ranked by *P*-value) are compared by best-match average (BMA), in which every term in either set is matched to its most similar counterpart in the other set and the matches are averaged. Wang BMA is primary because GO is redundant by construction: exact-match metrics treat terms as unrelated tokens, so a transferred set that recovers, say, “response to molecule of bacterial origin” where the truth contains “response to lipopolysaccharide” is scored as a complete miss despite reaching the same biological conclusion, and which of several near-synonymous terms crosses the significance threshold can be unstable; Wang BMA instead credits semantically close, non-identical matches [35] and is therefore robust to this redundancy. Exact-match metrics—precision, recall, F1 and the Jaccard index of the significant sets—were reported alongside, because they answer the complementary question of how many specific term identities are recovered or spurious, which is what exposes the recall–precision trade-off of reference annotation richness. Metrics were stratified by GO aspect (BP/MF/CC) and term IC, with 95% confidence intervals obtained by bootstrapping over gene lists (not experimental replicates); the complete per-list set-level results are provided in Table S2.

### Tolerance modeling

Per clade and aspect, mean Wang similarity was modeled as a four-parameter logistic in median ortholog identity *x*: Wang(*x*) = floor + (plateau − floor) / (1 + exp(−*k*(*x* − ID50))), fitted by constrained non-linear least squares (floor ≥0; plateau within [floor, observed maximum]; ID50 within the observed identity range; slope *k* > 0), giving a near-distance plateau, a far-distance floor and a half-collapse identity (ID50). A 95% confidence interval for ID50 was obtained from 1000 bootstrap resamples of the reference panel. Fits failing an identifiability criterion—boundary-pinned slope (*k* ≥ 5), bootstrap CI wider than 25 identity points, or R^2^ < 0.5—were flagged and a robust interpolated half-point reported instead (flagged fits appear as dashed curves in Fig. S2).

### Determinant analyses

The distance-versus-richness contrast used “equidistant” reference groups—references phylogenetically equidistant from a focal species but differing in annotation richness (mean experimental GO terms per gene)—together with multiple regression of set-level metrics on distance, richness, method, aspect and clade. Equidistant groups were designated *a priori* at panel-design time: the 19 flagged references (Table S1, column equidistant_group; marked in Fig. S1) form six groups, one per focal species, whose within-group spread in median ortholog identity is at most 4.8 percentage points and at most 1.8 points in five of the six groups—small relative to the ~40%–100% identity range of the full panels—while experimental annotation richness within a group varies 1.3- to 3.3-fold; residual within-group distance differences are additionally absorbed by the median-identity covariate in the regression. GO terms (biological process) were classified by ontology ancestry: a term was labeled taxon-specific if any ancestor was a lineage-variable root (developmental process, anatomical structure development, immune system process, behavior, multicellular organismal process, reproductive process, cell communication or signaling), conserved if any ancestor was a housekeeping root (metabolic process, translation, DNA or RNA metabolic process, cell cycle, generation of precursor metabolites and energy, or ribosome biogenesis), with taxon-specific taking precedence when both applied and otherwise other; the exact root GO identifiers are given in the deposited analysis code. The conserved-versus-specific effect was tested by ordinary least squares of Wang similarity on standardized ortholog identity, term category (reference level: taxon-specific) and their interaction, with clade as a covariate. To test whether the category contrast is confounded by orthology coverage, we additionally computed, for every synthetic BP list under every reference, the fraction of list genes with an RBH ortholog and the median amino-acid identity of the mapped genes (Table S3), and repeated the OLS with these as additional covariates. Whole-genome-duplication (WGD) effects were assessed from ortholog-map ambiguity (non-reciprocity and many-to-one rates) and from a fungi-internal comparison of post- versus pre-WGD references at matched distance.

### Orthogonal validation

Because the benchmark’s gold standard is itself GO-based, an apparent fidelity decline could in principle reflect how annotation happens to be distributed across species rather than genuine biological divergence; a genuine signal, however, should co-vary with functional-divergence measures computed without any GO input. As such a GO-independent check, expression conservation between focal and reference species was computed from Bgee [36] as the median, across one-to-one orthologs, of the Spearman correlation of their expression profiles over shared anatomical structures, a measure of cross-species transcriptome conservation [20, 37]. Because cross-species anatomy mapping is reliable within but not across phyla, expression-conservation references were restricted to within-phylum species; cross-phylum references were excluded regardless of mappability, and the insect focal had no within-phylum Bgee-mappable reference (not estimable). Protein-interaction-network conservation (STRING; [38]) was evaluated as a secondary check (Fig. S3; Table S5).

### Null baselines

Two null models contextualize the Wang scores. First, a random-term-set baseline: per clade and aspect we drew 1000 pairs of random, size-matched term sets from the pool of terms enriched in the focal truth and computed their Wang best-match average, giving the similarity expected between unrelated sets (non-zero because any two GO-term sets share ancestors in the DAG); far-distance floors are read relative to this null (Fig. S4). Second, a random-orthology control: holding the borrowed reference genes fixed so that annotation density is preserved, we shuffled which focal gene received which reference gene’s GO, re-ran enrichment and scored Wang against the truth (five shuffles per reference), testing whether correct orthology carries signal beyond density (Fig. S5).

## Results

### Cross-species GO-transfer fidelity declines and collapses with divergence

In every focal species, set-level fidelity was high when the reference was a close relative and fell as median ortholog identity decreased (Fig. 1A; dashed vertical lines mark each focal species’ clade boundary, defined as the identity of its phylogenetically nearest out-of-clade reference). The fitted decline was monotonic in all six clades and all three GO aspects, but its onset differed among focal species, and individual references scatter around the fits: the constrained logistic explained most of the reference-level variance for biological process (R^2^ 0.63–0.98 across clades, median 0.94) but less for cellular component (R^2^ 0.27–0.94), consistent with CC being the noisiest aspect (per-clade fit statistics in Table S6). Logistic tolerance curves gave a half-collapse identity (ID50, biological process) of ~40% for fly, yeast and zebrafish, ~55%–56% for the two plants, and ~59% for mouse (Fig. 1B; per-clade logistic fits in Fig. S2 and full parameters in Table S6). ID50 must be read together with the far-distance floor, however: mouse reaches its half-point at high identity yet retains a high floor (0.58), so its transfer never degrades far, whereas rice both collapses early and falls deepest (floor 0.30). Yeast combined a low ID50 with the highest floor (0.71), staying tolerant and declining only shallowly. Floors must be read against a null, however: because Wang similarity between random GO-term sets is itself substantial (~0.46 for BP, owing to shared DAG ancestors), mammal, insect and fungi (and marginally *Arabidopsis*) retained floors above this null, whereas fish and rice decayed to or below it—a genuine collapse to noise rather than a residual plateau (Fig. S4; Table S7). Molecular function and cellular component showed the same clade ordering at lower absolute similarity, with cellular component the noisiest aspect; their ID50 and floor values are reported alongside BP in Fig. 1B (Fig. 1A and B; Fig. S6).

**Figure 1:**
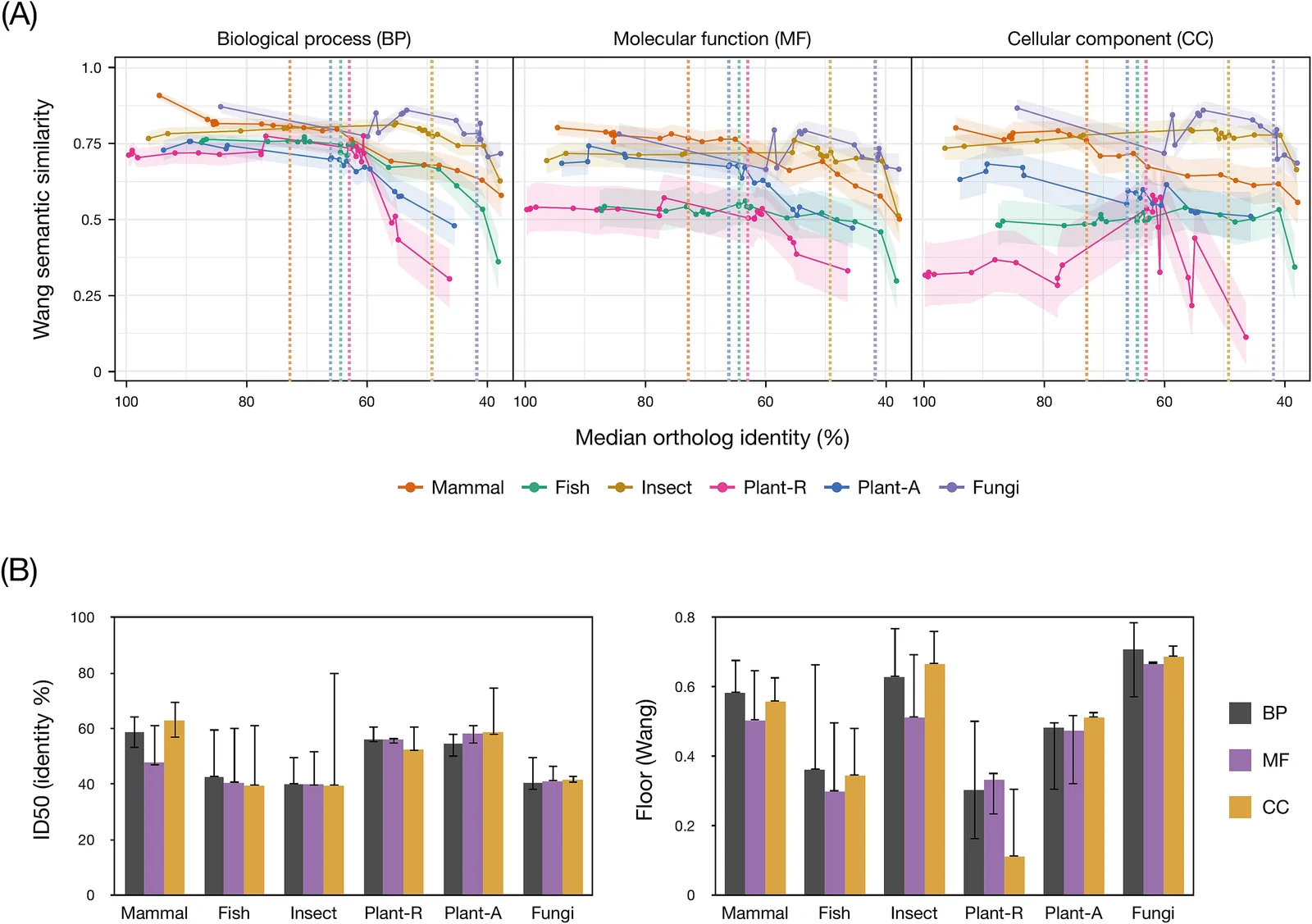
Divergence-tolerance of cross-species GO-transfer enrichment. (A) Set-level fidelity—Wang semantic similarity between the transferred and the experimental-truth enriched term sets (reciprocal-best-hit orthologs, all-evidence reference annotation, synthetic gene lists)—versus median ortholog identity (axis reversed; left = closer), for biological process (BP), molecular function (MF) and cellular component (CC). One line per focal species (Mammal, mouse; Fish, zebrafish; Insect, fly; Plant-R, rice; Plant-A, *Arabidopsis*; Fungi, yeast). Shaded bands = 95% bootstrap confidence intervals across gene lists (not experimental replicates). Dashed vertical lines mark, per focal species, the clade boundary—the median ortholog identity of the phylogenetically nearest out-of-clade reference (identical across the three aspect panels, since identity is a property of the reference, not of the GO aspect); note that taxonomy and identity are not perfectly collinear—one within-clade reference each for fish (grass carp) and insect (pea aphid) lies beyond this line, which is why the divergence axis uses identity rather than taxonomy. (B) Half-collapse identity (ID50, the % identity at which the curve reaches the midpoint between its near-distance plateau and far-distance floor) and the floor (residual Wang at maximum divergence), per focal species and GO aspect (BP, MF, CC). Error bars = 95% bootstrap confidence intervals from 1000 resamples of the reference panel, bootstrapping the same estimator that produced each reported value (constrained logistic where identifiable, robust interpolation otherwise); intervals are asymmetric, and for the interpolation-based floors (the minimum across references) the lower bound coincides with the estimate, giving one-sided upward intervals. A higher ID50 means the curve collapses at higher identity (less tolerant) and must be read together with the floor.

### Reference annotation richness trades recall for precision

Using equidistant reference groups to hold phylogenetic distance fixed while varying annotation richness, a richer reference raised recall (Spearman ρ = 0.62, *P* = .005) but lowered precision (ρ = −0.56, *P* = .012; n = 19 equidistant references) (Fig. 2). Multiple regression with clade included gave the same signs within every clade, so the trade-off is taxon-independent and not an artifact of pooling. In practice a better-annotated but equally distant reference recovers more truly enriched terms while also calling more spurious ones, leaving net F1 little changed. This quantifies the proximity-versus-annotation tension that Primmer et al. raised but did not measure [2].

**Figure 2:**
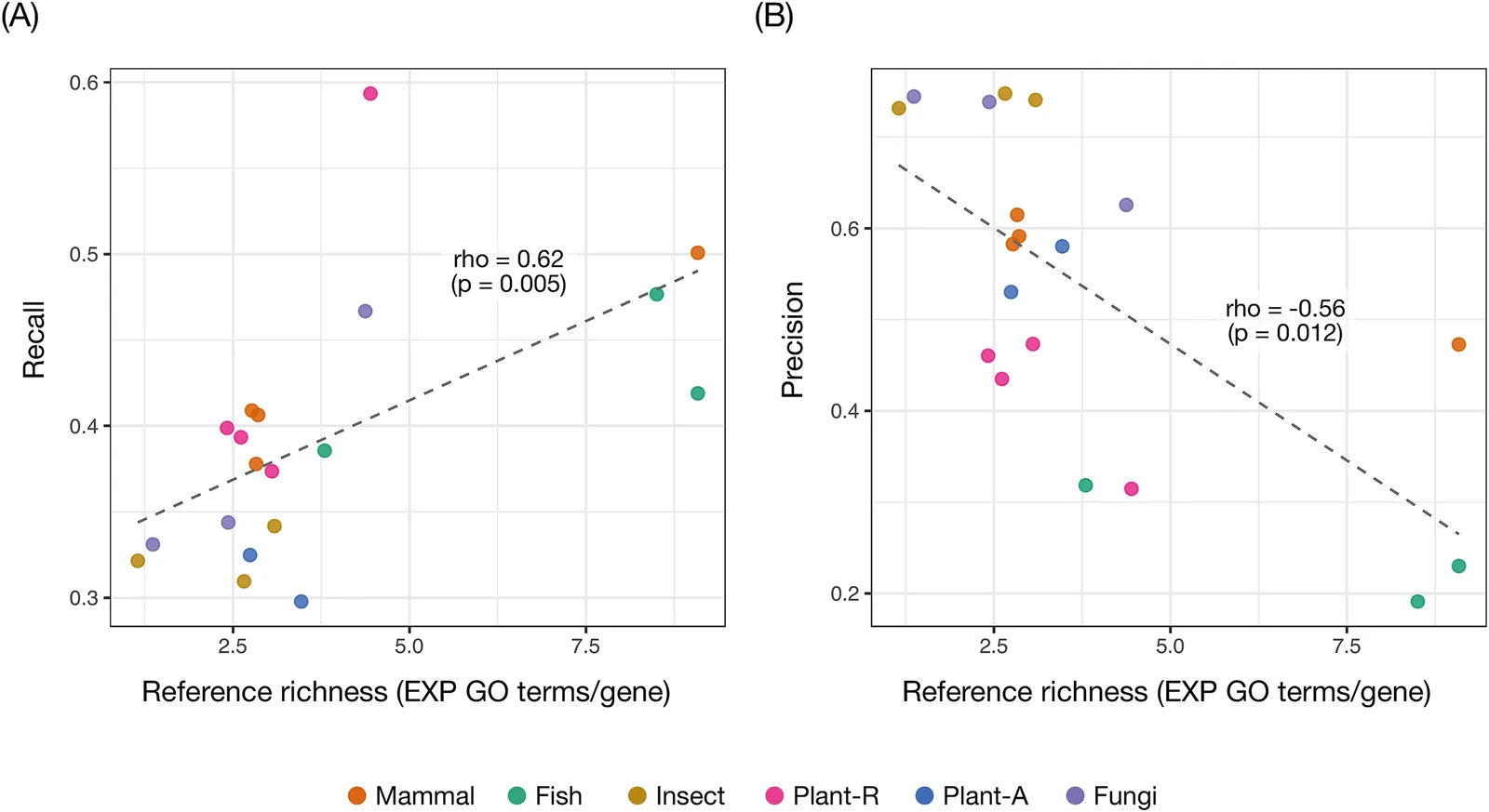
Reference annotation richness trades recall for precision. Among equidistant reference groups (phylogenetic distance held fixed within each focal species), set-level recall (A) and precision (B) versus reference annotation richness (mean experimental GO terms per gene); BP, reciprocal-best-hit, synthetic lists. Each point = one reference; dashed line = linear fit; Spearman ρ and p are pooled across the 19 equidistant references (multiple regression with clade controlled gives the same signs within each clade).

### Conserved processes transfer far; taxon-specific processes do not

Splitting GO terms into conserved versus taxon-specific functions, conserved-process fidelity stayed high out to distant references while taxon-specific fidelity collapsed, in all six clades (Fig. 3A). Pooled regression gave a large conserved main effect (0.19, *P* = 8 × 10^−283^) and a significant interaction by which conserved processes also decayed more slowly with distance (−0.08, *P* = 6 × 10^−56^). This category effect is not an artifact of differential orthology coverage: conserved lists do map somewhat better (mean RBH mapping rate 0.73 versus 0.63 for taxon-specific lists; median identity of list genes 67.4 versus 65.2%; Table S3), but adding the per-list mapping rate—or both mapping rate and gene-level identity—as covariates leaves the conserved main effect dominant and highly significant (+0.15, *P* = 3 × 10^−170^, and + 0.15, *P* = 7 × 10^−182^, respectively). Stratifying by term information content (Fig. 3B) sharpened the picture: shallow (general) terms were recovered with F1 that declined with distance, whereas deep (specific) terms were barely recovered at any distance (F1 ≈ 0). Borrowed annotation therefore supports broad, conserved conclusions across large evolutionary gaps but cannot resolve specific or lineage-restricted functions; the same pattern held in all six focal species tested, spanning animals, plants and fungi.

**Figure 3:**
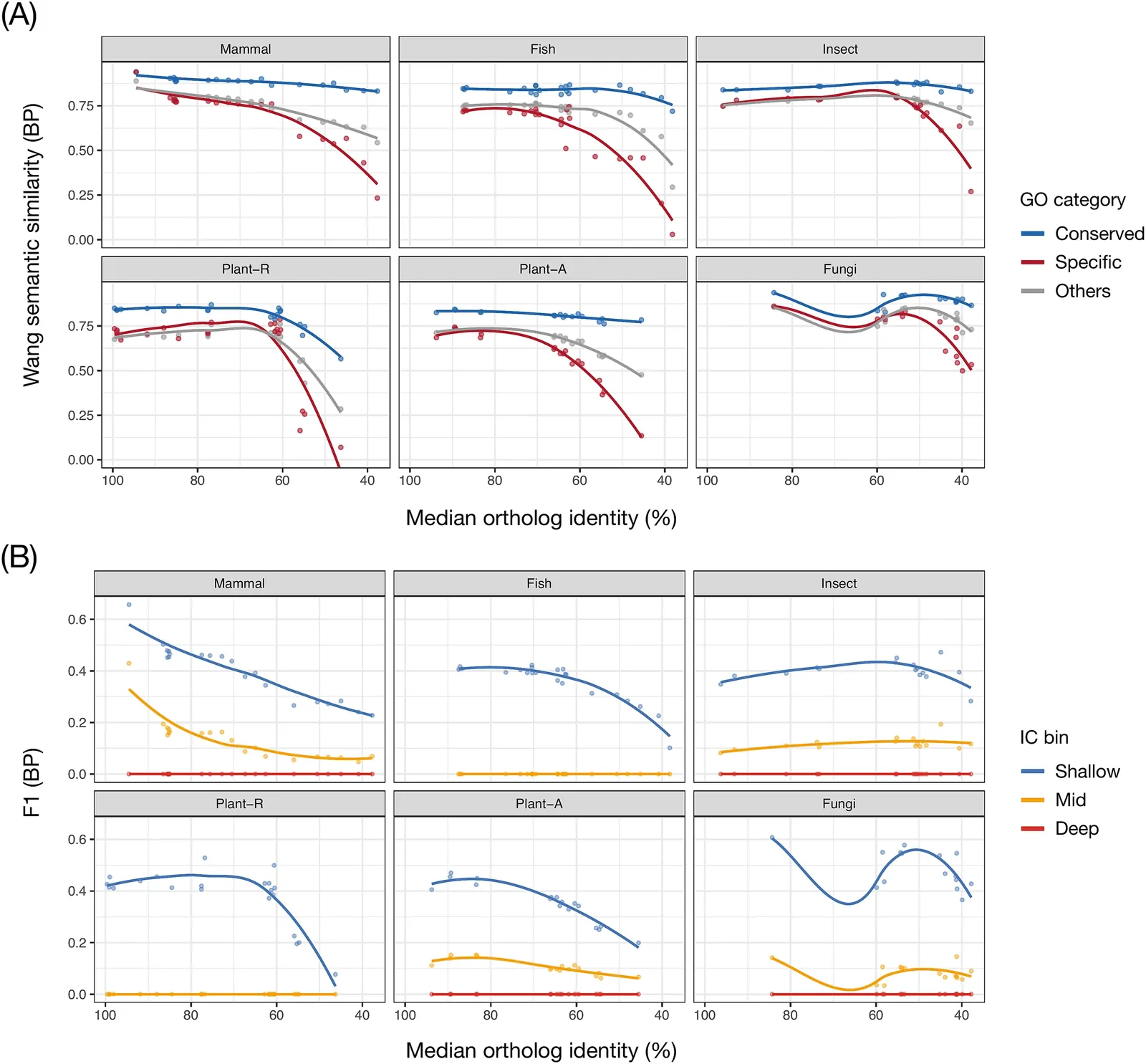
Conserved processes transfer far; taxon-specific processes do not. (A) Wang similarity versus median ortholog identity for conserved (metabolism, translation, DNA processing), taxon-specific (development, immunity, behavior) and other GO terms, per focal species (BP, reciprocal-best-hit). (B) Set-level F1 stratified by term information content (IC) bin—shallow (general), mid, deep (specific); deep/specific terms are barely recovered at any distance. F1 (rather than Wang) is shown because semantic similarity is not defined per single list within an IC bin.

### Orthology method and genome architecture

The two plants dropped markedly more under strict RBH than under best-hit, whereas animals and fungi were largely insensitive to the choice (Fig. 4A). Quantified as an RBH penalty (Fig. 4B), RBH raised precision and lowered recall in every clade, but the net Wang penalty was largest for the two plants (−0.02 to −0.04). eggNOG-OG membership behaved like best-hit, preserving recall while staying orthology-grounded (mean recall 0.386 versus 0.390; precision 0.584 versus 0.581)—so in paralog-rich genomes eggNOG or best-hit is preferable to strict RBH (Fig. 4C; per-reference values in Fig. S7 and Table S8). The *a priori* hypothesis that paralog-rich plants would show steeper tolerance curves was not supported as curve steepness; instead, plant best-hit maps carried the most ortholog ambiguity (mean non-reciprocity 0.41 for plants versus 0.38 for fish and 0.31 for mammal, despite plant references sitting at comparable or higher sequence identity), and the WGD effect surfaced as sensitivity to the orthology method rather than as faster decay. A fungi-internal contrast of post- versus pre-WGD references showed no extra penalty at matched distance (Fig. S8), consistent with yeast’s ancient WGD having been largely resolved by gene loss. WGD thus degrades GO transfer only when ohnolog retention is high (recent WGD, as in plants), and chiefly through method choice.

**Figure 4:**
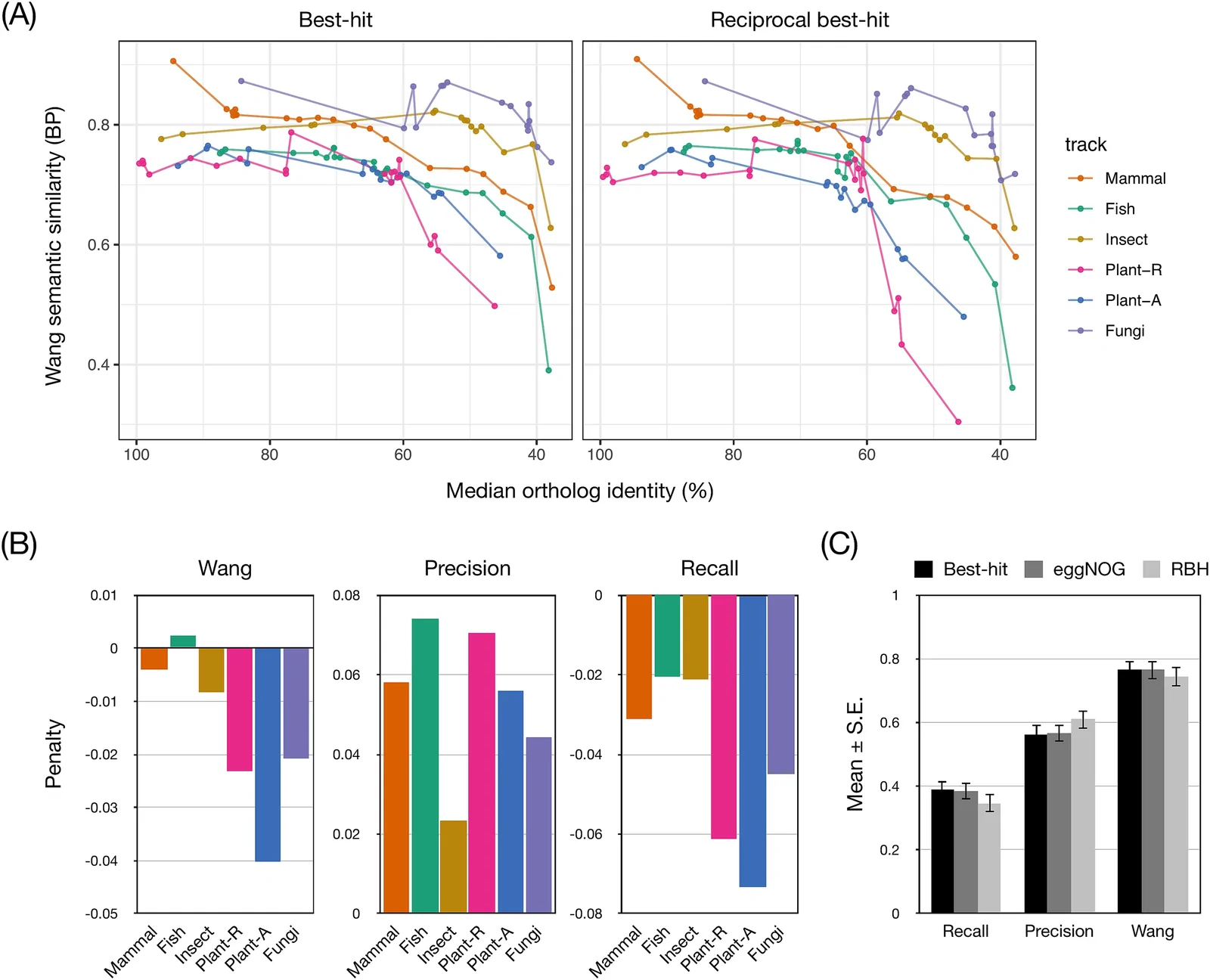
Orthology method and whole-genome duplication. (A) Tolerance curves under best-hit versus reciprocal-best-hit (RBH) orthologs (BP). (B) RBH penalty (RBH − best-hit) in Wang, precision and recall per focal species (negative Wang = RBH worse). (C) Mean recall, precision and Wang by orthology method (best-hit, eggNOG-OG, RBH) across the 24 references carrying all three methods; error bars = standard error across references.

### The tolerance signal is real, and real-world use is capped by annotation sparsity

A GO-based benchmark faces a circularity concern: an apparent fidelity decline could reflect how annotation happens to be distributed across species rather than genuine functional divergence. If that were the case, the Wang fidelity score would have no reason to co-vary with a measure of functional divergence computed purely from expression data. We therefore correlated GO-transfer fidelity with expression conservation across references (Fig. 5A; Table S9). Across vertebrate references, expression conservation predicted GO-transfer fidelity (mouse ρ = 0.60, n = 13; zebrafish ρ = 0.77, n = 6; pooled ρ = 0.62, *P* = .005, n = 19): references whose orthologs have diverged more in expression also yield worse GO transfer, so the measured decline tracks biological divergence rather than annotation idiosyncrasy. This validation establishes that the fidelity signal is biologically grounded; it does not by itself certify the accuracy of any individual enrichment result. As a second, independent control, correct ortholog transfer far exceeded a density-matched random-orthology baseline (mean Wang ≈ 0.7–0.9 versus ≈ 0.2 across references), converging toward it only at the deepest references—confirming that the transferred signal reflects orthology rather than annotation density and that it genuinely vanishes at extreme divergence (Fig. S5; Table S10). This validation was feasible only within phyla and only for the two vertebrate focals, because cross-species expression comparison is unreliable across phyla and the insect focal had no within-phylum expression-mappable reference (Methods). When we applied the benchmark to real DEG sets, real lists scored well below synthetic ones—but this gap was a property of the gold standard, not of transfer. At matched list size, real lists still fell far below synthetic (e.g. 0.19 versus 0.72 for lists <100 genes; Fig. 5B; list-size × IC robustness in Fig. S9 and Table S11), and the real-DEG score tracked the focal species’ experimental-truth coverage (Spearman ρ = 0.94, *P* = .017, n = 6 focal species; Fig. 5C). Where a focal species’ own experimental annotation is sparse (rice, 2.4% coverage), too few of a real list’s genes carry truth to validate the result—a measurement ceiling, not a transfer failure. Synthetic lists therefore remain the valid instrument for the tolerance curves, while real DEGs document the real-world coverage limit.

**Figure 5:**
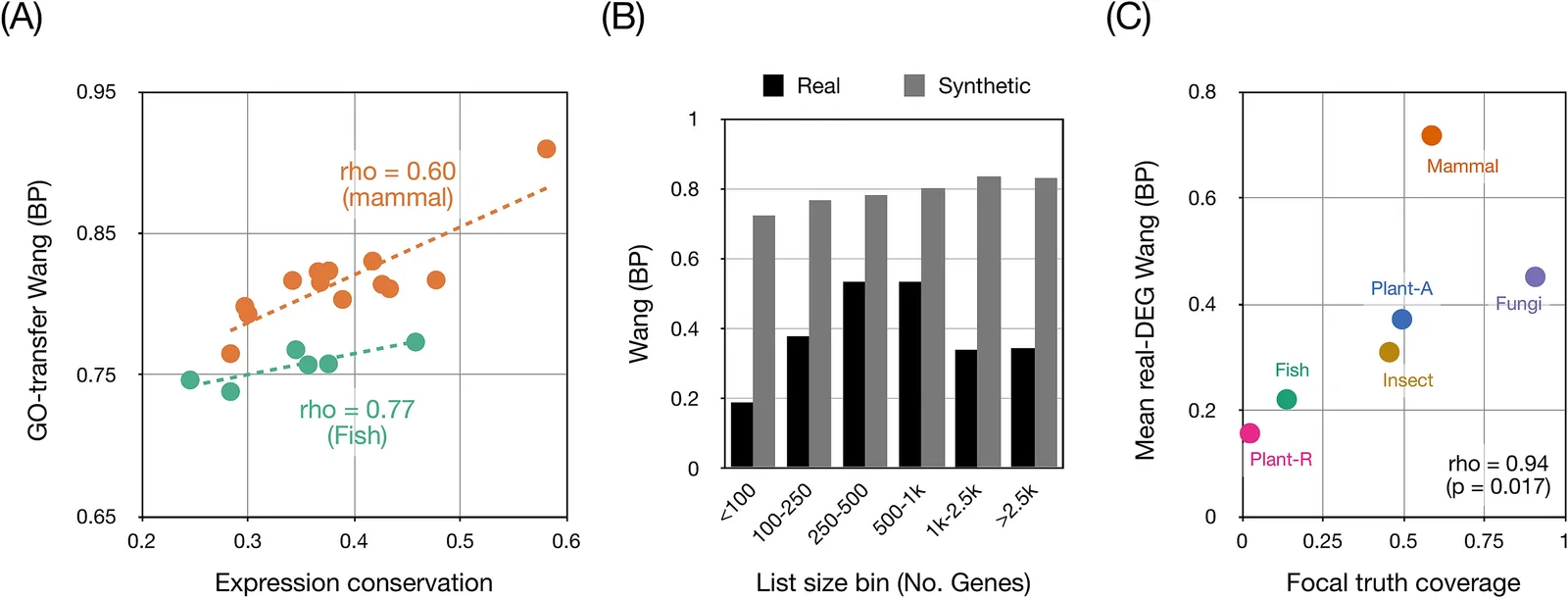
The tolerance signal is real, and real-world use is capped by annotation sparsity. (A) GO-transfer fidelity versus a GO-independent measure—Bgee expression conservation—for the two vertebrate focal species (within-phylum references only; per-track Spearman ρ shown). (B) Mean Wang by gene-list size bin for real versus synthetic lists; real < synthetic at every matched size (bars = mean ± SE across reference × list), excluding a size artifact. (C) Mean real-DEG Wang versus the focal species’ experimental-truth coverage (Spearman ρ shown).

### A practical reliability guide

Distilling the benchmark, Table 1 reports the mean transferred-versus-truth Wang similarity for each focal species, reference-distance band (near >70%, mid 55%–70%, far <55% median ortholog identity) and GO aspect, with the number of supporting references (full per-aspect values for every distance band in Table S12). The table makes the operating rules concrete: borrowing is reliable for conserved-rich animal and fungal clades even at moderate distance, marginal for plants at mid distance, and untrustworthy for plants and for cellular component at far distance. Reading each cell against its null row sharpens this further: the heuristic thresholds are anchored to the BP/MF null (0.43–0.51), whereas the CC null is higher (0.54–0.64), so several CC cells—for rice, at every distance band (e.g. 0.33 at near distance versus a null of 0.64)—fall at or below chance, quantitatively substantiating the warning above. Cells supported by few references (e.g. Fungi at near distance, n = 1) should be read with caution (Table 1).

**Table 1:** Practical reliability guide. Mean transferred-versus-truth Wang semantic similarity by focal species, reference-distance band and GO aspect (reciprocal-best-hit orthologs, all-evidence reference annotation), with the number of supporting reference species (*n*) and 95% cluster-bootstrap confidence intervals in brackets (10 000 resamples of the supporting references; the single-reference cell, fungi/near, is marked n = 1 and carries no CI). Each focal species’ random-term null row gives the Wang similarity expected between the truth-enriched set and a size-matched random term set; cells should be read against this chance level, which is aspect-dependent (0.43–0.51 for BP and MF, 0.54–0.64 for CC). Distance bands: near, >70%; mid, 55%–70%; far, <55% median ortholog identity. The heuristic flags (≥0.60 reliable, 0.45–0.60 caution, <0.45 unreliable) are anchored to the BP/MF null band; cellular-component cells are best judged against their own, higher null row. Cells with small *n* should be interpreted cautiously.

Focal	Distance	n	BP	MF	CC
Mammal	near	11	0.82 [0.81–0.84]	0.78 [0.77–0.79]	0.77 [0.76–0.78]
Mammal	mid	4	0.76 [0.72–0.80]	0.73 [0.69–0.77]	0.69 [0.66–0.71]
Mammal	far	5	0.65 [0.62–0.68]	0.62 [0.56–0.66]	0.62 [0.59–0.64]
Mammal	random-term null		0.43	0.44	0.54
Fish	near	9	0.76 [0.76–0.77]	0.53 [0.53–0.54]	0.49 [0.49–0.50]
Fish	mid	8	0.73 [0.71–0.75]	0.54 [0.53–0.55]	0.51 [0.50–0.52]
Fish	far	5	0.59 [0.49–0.66]	0.47 [0.41–0.51]	0.49 [0.44–0.52]
Fish	random-term null		0.48	0.46	0.61
Insect	near	5	0.79 [0.78–0.80]	0.71 [0.70–0.72]	0.76 [0.74–0.77]
Insect	mid	2	0.82 [0.81–0.82]	0.74 [0.72–0.76]	0.79 [0.79–0.80]
Insect	far	9	0.76 [0.73–0.79]	0.69 [0.65–0.72]	0.77 [0.74–0.78]
Insect	random-term null		0.45	0.46	0.57
Plant-R	near	10	0.72 [0.71–0.74]	0.54 [0.53–0.55]	0.33 [0.31–0.34]
Plant-R	mid	9	0.68 [0.61–0.73]	0.50 [0.47–0.52]	0.46 [0.38–0.54]
Plant-R	far	2	0.39 [0.30–0.43]	0.36 [0.33–0.39]	0.33 [0.11–0.44]
Plant-R	random-term null		0.51	0.47	0.64
Plant-A	near	5	0.74 [0.73–0.76]	0.71 [0.69–0.73]	0.66 [0.64–0.67]
Plant-A	mid	9	0.67 [0.65–0.69]	0.64 [0.61–0.66]	0.57 [0.55–0.59]
Plant-A	far	3	0.55 [0.48–0.58]	0.51 [0.47–0.54]	0.52 [0.51–0.52]
Plant-A	random-term null		0.45	0.43	0.55
Fungi	near	1	0.87 [n = 1]	0.78 [n = 1]	0.87 [n = 1]
Fungi	mid	3	0.80 [0.77–0.85]	0.71 [0.67–0.80]	0.77 [0.72–0.85]
Fungi	far	11	0.79 [0.76–0.82]	0.73 [0.70–0.75]	0.79 [0.75–0.82]
Fungi	random-term null		0.48	0.47	0.59

## Discussion

We benchmarked, for the first time at the level practitioners use, how far cross-species GO-enrichment can be pushed before it fails. The result is a set of operating rules rather than a single cutoff. Fidelity declines monotonically with divergence—collapsing to chance at the clade boundary in some panels, plateauing well above it in others—and how soon and how far it falls depends on the focal species, the reference, and the type of function. For routine use this translates into concrete guidance: prefer a better-annotated reference even at the same distance when the question concerns conserved processes (richness buys recall); distrust taxon-specific and cellular-component conclusions once references fall below the mid-distance band; use eggNOG-OG or best-hit rather than strict reciprocal-best-hit for paralog-rich genomes such as plants; and consult the focal-species × distance reliability table (Table 1) before trusting a borrowed-GO result. These steps are summarized as a step-by-step decision workflow (Fig. 6).

**Figure 6:**
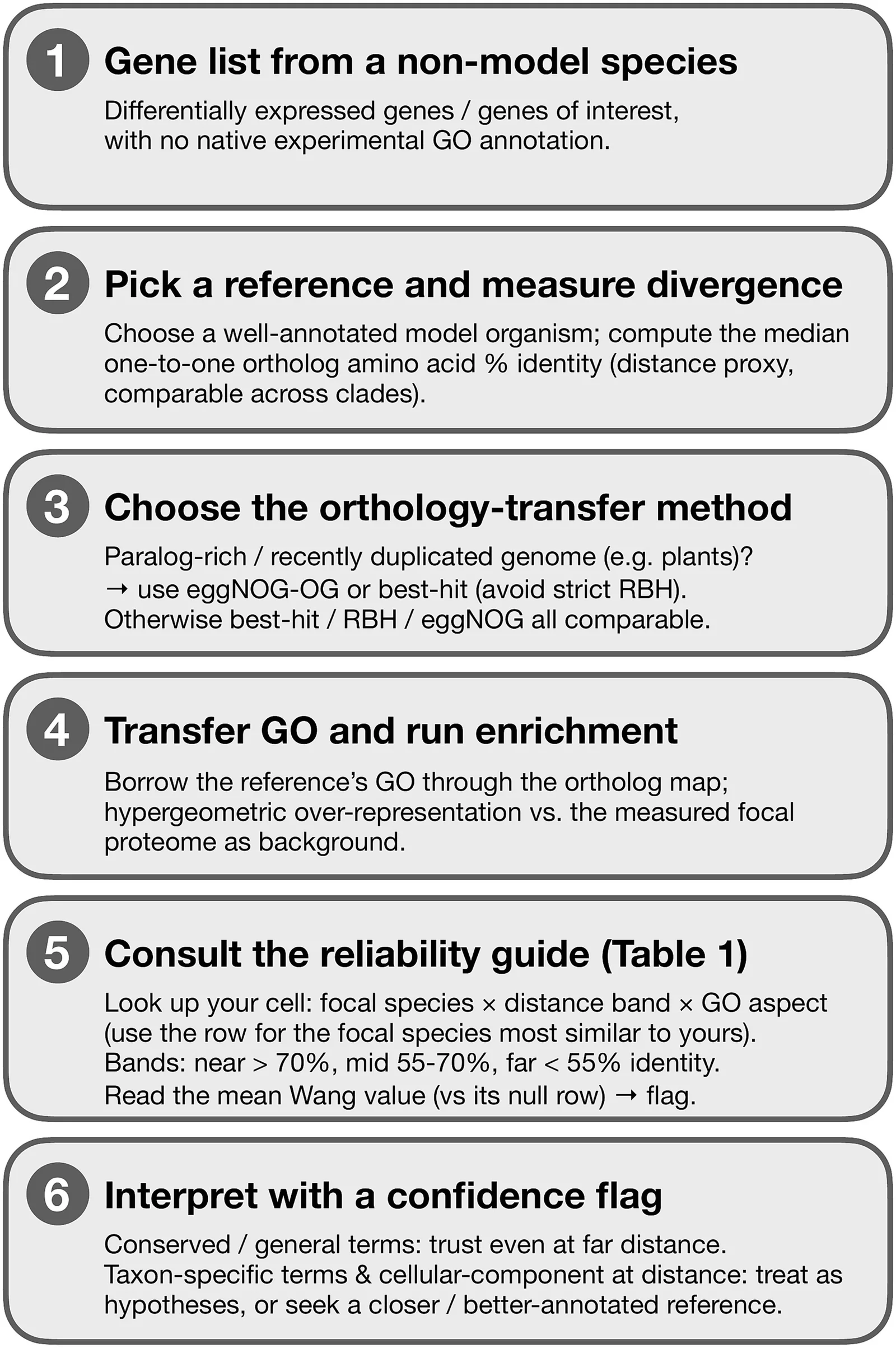
A decision workflow for applying cross-species GO-transfer enrichment to a non-model species. Six-step protocol: (i) begin with the non-model gene list; (ii) choose a well-annotated reference and quantify divergence as the median one-to-one ortholog amino-acid % identity; (iii) select the orthology-transfer method—for paralog-rich or recently duplicated genomes (e.g. plants) use eggNOG-OG or best-hit and avoid strict reciprocal-best-hit (Fig. 4); (iv) transfer the reference GO through the ortholog map and run hypergeometric over-representation against the measured focal proteome; (v) look up the focal-species × distance-band × GO-aspect cell in the reliability guide (Table 1, using the row for the focal species most similar to the species at hand; distance bands: near >70%, mid 55%–70%, far <55% median ortholog identity); and (vi) interpret with the resulting confidence flag. Reliability flag on the mean transferred-versus-truth Wang similarity, read against the focal species’ random-term null row in Table 1: ≥0.60 reliable, 0.45–0.60 caution, <0.45 unreliable. Conserved and general terms transfer reliably even at far distance, whereas taxon-specific terms and cellular-component annotations at distance should be treated as hypotheses or re-tested with a closer or better-annotated reference.

Our findings recast the long-running ortholog-conjecture debate [21–23, 39, 40] in practical terms. Those studies measured functional similarity between individual gene pairs; we measure whether the *enrichment result* survives, which is what a biologist obtains. At the set level the conjecture “holds enough”: conserved-process enrichment transfers reliably across large evolutionary gaps, whereas specific and lineage-restricted functions do not transfer at all, so its practical validity depends on the type of function. This also sharpens the trade-off that Primmer et al. articulated but did not quantify [2]: a closer reference reduces orthology error, a richer reference recovers more true terms but adds spurious ones, and the two pulls can now be balanced with numbers.

The whole-genome-duplication result runs against an intuitive but incorrect expectation. Paralog-rich plants, shaped by recurrent angiosperm polyploidy [41, 42], do not show steeper tolerance curves; rather, duplication inflates ortholog-mapping ambiguity and thereby makes the result sensitive to the orthology method, an effect that is invisible if one only inspects the curves. Other focal lineages carry their own duplications—the teleost-specific event in zebrafish and the two ancestral vertebrate rounds [43, 44]—yet showed no comparable method sensitivity, pointing to ohnolog retention rather than WGD per se as the driver [45, 46]. Because yeast’s ancient WGD [47, 48]—largely resolved by gene loss—carries no such penalty, WGD harms transfer only when ohnolog retention is high.

Several limitations bound these conclusions. First, with one focal species per clade (two for plants), focal-species effects and clade-level effects are confounded by design; the two plant panels illustrate this—far-distance BP fidelity is 0.39 for rice but 0.55 for *Arabidopsis*—so measured tolerance is a property of a focal species and its reference panel rather than of a clade in the abstract, and the rows of Table 1 should be read as focal-species-specific starting estimates, not clade-wide constants. Second, the gold standard is itself GO-based; we mitigated the resulting circularity by restricting truth to experimental evidence and by an orthogonal, GO-independent expression validation, but that validation was feasible only within vertebrate phyla, since cross-species expression comparison breaks down across phyla [49] and the insect focal lacked a within-phylum mappable reference. The reliability thresholds are anchored to the random-term null rather than arbitrary, but they remain descriptive summaries of this benchmark: they have not been validated on independent focal species or by prospective testing, and they are offered alongside the underlying numeric values and per-focal null rows (Table 1) so that readers may apply their own criteria; prospective validation on additional focal species is a natural next step. Synthetic gene lists, though derived from the focal annotation, provide the controlled signal needed to chart the curves and are independent of the transferred annotation under test; real DEG validation is limited by the sparsity of experimental annotation rather than by transfer. Finally, our scope is eukaryotic: prokaryotes, where horizontal gene transfer and operonic organization undermine the distance-to-function premise, would behave differently and warrant separate study.

**Key Points**

- Cross-species GO-enrichment fidelity decays monotonically with phylogenetic distance; in some focal species (fish, rice) it collapses to chance at the clade boundary, in others (insect, fungi, mammal) it retains a usable floor (ID50 ~ 40–59% median ortholog identity).
- Reference choice is a recall–precision trade-off: a richer (better-annotated) reference recovers more true terms but adds spurious ones, independent of phylogenetic distance.
- Conserved (metabolic, translational) functions transfer reliably to distant references; taxon-specific functions (development, immunity) do not—consistent in all six focal species tested (three eukaryotic kingdoms).
- Whole-genome duplication harms transfer only with high ohnolog retention (plants), and chiefly through sensitivity to the orthology-assignment method (strict reciprocal-best-hit over-penalizes paralog-rich genomes).
- A practical focal-species × distance reliability table lets users judge, before analysis, whether borrowed-GO enrichment can be trusted for their species and GO aspect.

## Conclusions

Borrowing Gene Ontology annotation from a model organism to interpret a non-model species is a near-universal practice whose limits have never been mapped. Across three eukaryotic kingdoms we show that it works, but only conditionally: conserved functions transfer across large divergences while specific functions do not, reference annotation richness trades recall for precision, paralog-rich genomes demand careful orthology methods, and reliability is quantitative but specific to each focal species and its reference panel. The accompanying reliability guide turns these findings into a practical tool for the many studies that depend on borrowed annotation.

## Supplementary Material

Supplementary_materials_bbag622

## Data Availability

All raw data derive from public resources (UniProt, the GO Annotation database, Bgee, the EBI Expression Atlas and TimeTree). Supplementary Tables S1–S12 are provided in Supplementary_Tables.xlsx (Table S2, the full per-list set-level results, is supplied as a separate Excel file (Table_S2.xlsx) owing to size). The processed result tables, figure-generation scripts and the full reproducible analysis pipeline are archived at Zenodo (https://doi.org/10.5281/zenodo.20758129) and available on GitHub (https://github.com/tjdrnjsqpf/cross-species-GO-benchmark).
