# Supplementary material for: How far can you borrow? Divergence limits of cross-species Gene Ontology enrichment in non-model organisms
Source: Brief Bioinform. 2026 Nov 9;27(6):bbag622. doi: 10.1093/bib/bbag622 (PMC13747021; doi:10.1093/bib/bbag622)
Supplement: Supplementary file 1 [file supplementary_materials_bbag622.zip › Supplementary_Information_bbag622.docx]

**Supplementary Information**

How far can you borrow? Divergence limits of cross-species Gene Ontology enrichment in non-model organisms

Sung-Gwon Lee and Chungoo Park

**Figure S1.** Reference panels of the six focal species along the divergence axis. Each panel shows the full reference set of one focal species, labeled with full scientific names and TimeTree divergence times (My). Points mark the median one-to-one ortholog amino-acid identity; thick bars span the interquartile range and thin lines the 5th–95th percentile of the underlying per-ortholog identity distribution. Diamonds mark the equidistant richness-contrast groups used for the distance-versus-richness analysis (Fig 2; Methods).

*Alt text: Six dot plots, one per focal species, listing reference species by scientific name and showing each reference's median ortholog identity with its interquartile range along a reversed identity axis; diamonds mark the equidistant reference groups.*

**Figure S2.** Logistic tolerance fits (biological process) per focal species with ID50 markers (dotted vertical lines). Dashed curves = fits failing the identifiability criterion (interpolated half-point used).

*Alt text: Line plot of Wang similarity versus median ortholog identity with fitted logistic curves for six focal species; dashed curves indicate non-identifiable fits and dotted vertical lines mark ID50 values.*

**Figure S3.** STRING network-conservation as a secondary, GO-independent check (experiments + co-expression channels). Per-track correlation with GO-transfer fidelity is weak and inconclusive owing to sparse STRING coverage in non-model references; Bgee expression conservation (Fig. 5A) is the primary orthogonal validation.

*Alt text: Five scatter panels correlating STRING network conservation with GO-transfer fidelity per focal species; correlations are weak and inconclusive.*

**Figure S4.** Far-distance tolerance floor read against a random-term null (biological process). For each clade, the fitted far-distance floor (coloured) beside the Wang similarity expected between two random size-matched GO-term sets drawn from the enrichable term pool (truth vs random; grey; 1,000 pairs). Because Wang similarity is non-zero for unrelated GO sets (~0.46 for BP), floors are meaningful only above this null: mammal, insect and fungi (and marginally Arabidopsis) retain residual signal, whereas fish and rice decay to or below the null (numbers = floor − null). See Table S7.

*Alt text: Bar chart comparing each clade's far-distance floor with its random-term null; mammal, insect and fungi floors exceed the null while fish and rice fall at or below it.*

**Figure S5.** Correct ortholog transfer versus a density-matched random-orthology control (biological process). Per reference, Wang similarity of the real transfer (coloured) and of a control in which the borrowed reference genes are held fixed but their assignment to focal genes is shuffled (grey, mean ± SD over five shuffles), against median ortholog identity. Correct orthology greatly exceeds the random control across the near-to-mid range and converges toward it only at the deepest references, confirming that the transferred signal reflects orthology rather than annotation density. See Table S10.

*Alt text: Six panels showing Wang similarity of correct ortholog transfer versus a density-matched random-orthology control across reference identity; correct transfer greatly exceeds the control except at the most distant references.*

**Figure S6.** Divergence-tolerance measured by set-level recall (complementary to Wang similarity in Fig. 1A), for biological process (a), molecular function (b) and cellular component (c). Lines = focal species; shaded = 95% bootstrap confidence interval across synthetic gene lists.

*Alt text: Three line plots of set-level recall versus median ortholog identity for biological process, molecular function and cellular component, one line per focal species with confidence bands.*

**Figure S7.** Per-reference precision–recall by orthology method (best-hit, eggNOG-OG, RBH); grey lines link the same reference across methods. RBH shifts each reference toward higher precision and lower recall.

*Alt text: Scatter plot of per-reference precision versus recall under three orthology methods; grey lines link the same reference and show reciprocal-best-hit shifting each reference toward higher precision and lower recall.*

**Figure S8.** Fungi-internal paralog-mapping ambiguity for post-WGD versus pre-WGD references (raw; distance not controlled — pre-WGD references are also more distant). No extra penalty remains after distance control (Results).

*Alt text: Two bar charts of ortholog-mapping ambiguity (non-reciprocity and many-to-one rates) for post- versus pre-whole-genome-duplication fungal references.*

**Figure S9.** Synthetic-list Wang similarity versus list size, colored by term information content (IC). Large synthetic lists remain high partly because large GO terms are low-IC; the real-vs-synthetic gap (Fig. 5B) persists across the size range.

*Alt text: Scatter plot of synthetic-list Wang similarity versus list size on a log scale, colored by term information content; large lists remain high because large GO terms are low-information-content.*
